# The soybean experiment ‘1000 Gardens’: a case study of citizen science for research, education, and beyond

**DOI:** 10.1007/s00122-018-3134-2

**Published:** 2018-07-03

**Authors:** Tobias Würschum, Willmar L. Leiser, Felix Jähne, Kristina Bachteler, Martin Miersch, Volker Hahn

**Affiliations:** 1 State Plant Breeding Institute, University of Hohenheim, 70593 Stuttgart, Germany; 2 Taifun-Tofu GmbH, 79108 Freiburg, Germany

## Abstract

**Key message:**

Citizen science, an approach that includes normal citizens in scientific research, holds great potential also for plant sciences and breeding and can be a powerful research tool to complement traditional approaches.

**Abstract:**

Citizen science is an approach that includes normal citizens in scientific research, but has so far not been exploited by the various disciplines in plant sciences. Moreover, global threats challenge human well-being and science can provide solutions, but needs to leave the ivory tower in the mind of the broader public. In 2016, we performed the ‘1000 Gardens—the soybean experiment’ citizen science project, that aimed at finding citizens in Germany who would grow soybean lines in their own gardens and evaluate them for a range of traits related to adaptation and agronomic performance. Here, we describe details of this project, i.e. the recruitment, performance, and compliance of the citizen scientists. A total of 2492 citizen scientists volunteered for the project, but through the high media coverage a much broader audience than just the participants was reached. Our 1000 Gardens project was successful in collecting a scientifically unique data set with heritabilities ranging up to 0.60 for maturity date or 0.69 for plant height. Our results suggest that the citizen science approach holds great potential also for plant sciences and can be a powerful research tool to complement traditional approaches. Our project was also successful in raising public awareness about the importance of plant breeding and in communicating key messages on the manifold benefits of legumes for a sustainable agriculture to a broader public. Thus, citizen science appears as a promising avenue to demonstrate the value of breeding and science to the general public by including normal citizens in scientific research.

**Electronic supplementary material:**

The online version of this article (10.1007/s00122-018-3134-2) contains supplementary material, which is available to authorized users.

## Introduction

Citizen science is an approach that at least in part involves citizens as highly respected partners in research (Silvertown 2009). The idea itself has a long tradition, as for decades the help of such non-professionals has proven to be an invaluable and extremely powerful tool for ornithology to survey population numbers and spread (Magurran et al. 2010). The earliest what we now call citizen science projects, is probably the Christmas Bird Count by the National Audubon Society that has been running in the USA since 1900. More recently, a few other disciplines have also adopted this approach, and it has, for example, been successfully used in ecology and environmental sciences to monitor invasive species or in astronomy, where it has recently led to the discovery of a novel brown dwarf (Silvertown 2009; Magurran et al. 2010; Worthington et al. 2012; Kuchner et al. 2017). Worthington et al. (2012) employed the citizen science approach in the Evolution MegaLab project that surveyed shell polymorphism in two banded snails across Europe with the aim to compare these data with historical records to detect evolutionary change.

Generally, citizen science projects are designed either to generate scientific data for research or for the educational benefit of the amateur volunteers, ideally for both. Citizen science projects could, however, have a far greater potential than purely as a research tool or for the education of the participants. An exciting project may serve as a vehicle to transport a range of messages beyond the particular research question of the project. It could provide a platform for the participants to share their experiences and give them the feeling of a collective action, which could spread and grow through the participants as multipliers. Most importantly, appropriately designed projects could arouse moral emotions, i.e. make even complex topics emotionally accessible and thus open up the general public to scientific arguments and their consequences.

One such challenge that requires global change is the establishment of sustainable agriculture. The United Nations declared 2016 as the International Year of Pulses (grain legumes), under the slogan ‘nutritious seeds for a sustainable future’ (www.fao.org/pulses-2016). In contrast to cereals, grain legumes are capable of symbiotic atmospheric nitrogen fixation and generally have several desirable attributes, as they enhance biodiversity in our agro-ecosystems, contribute to soil fertility, and benefit both humans and livestock due to their advantageous nutritional characteristics (Foyer et al. 2016). A reduction in meat consumption by shifting to grain legume products could substantially reduce the carbon footprint caused by the production of protein for human consumption. Soybean is the most important leguminous crop worldwide, but Europe is currently heavily dependent on soybean imports. Soybean was never a major crop in Germany, and its cultivation has only recently seen a revival with a strong relative increase in soybean acreage. This was mainly driven by political efforts to reduce the high dependency on plant protein imports, but also by the desire of consumers for genetically modified (GM)-free, regional products. While the majority of the domestic soybean production is used for animal feed, its use for human consumption, especially through tofu products, has seen a tremendous increase, owing to strong consumer trends for vegetarian or vegan food, as well as for novel and healthy products that diversify our food basket. Compared to the traditionally cultivated crops, however, soybean still has a rather negative public image, mainly as the broader public associates it with being genetically modified, grown in large monocultures, and contributing to the deforestation in South America, Asia, and elsewhere.

Except for ecological studies, the citizen science approach is not yet recognized as a research tool for plant sciences. In 2016, the University of Hohenheim and the Taifun-Tofu GmbH performed the ‘1000 Gardens—the soybean experiment’ citizen science project, that aimed at finding citizens who would grow and evaluate soybean lines in their own gardens. The objectives of the project were to (1) collect data for scientific analyses such as the genetics underlying the adaptation of soybean to more northern latitudes and (2) improve the public image of soybeans and highlighting the manifold advantages of soybeans and legumes in general for a sustainable (regional) agriculture as well as the health-associated advantages of grain legume-rich diets. While normal plant breeding trials are performed at only a few locations, this approach has enabled data to be collected on the performance of soybean genotypes at an unprecedented number of locations throughout Germany. This allows the identification of genotypes that are adapted to more northern latitudes or regions where soybean is not yet widespread. Here, we describe how we planned and executed the 1000 Gardens project and make suggestions on how to improve future citizen science projects, as we believe that this approach has great potential also for various disciplines in plant sciences, for research, breeding, education, and beyond.

## Materials and methods

### Recruitment of citizen scientists

**Fig. 1:**
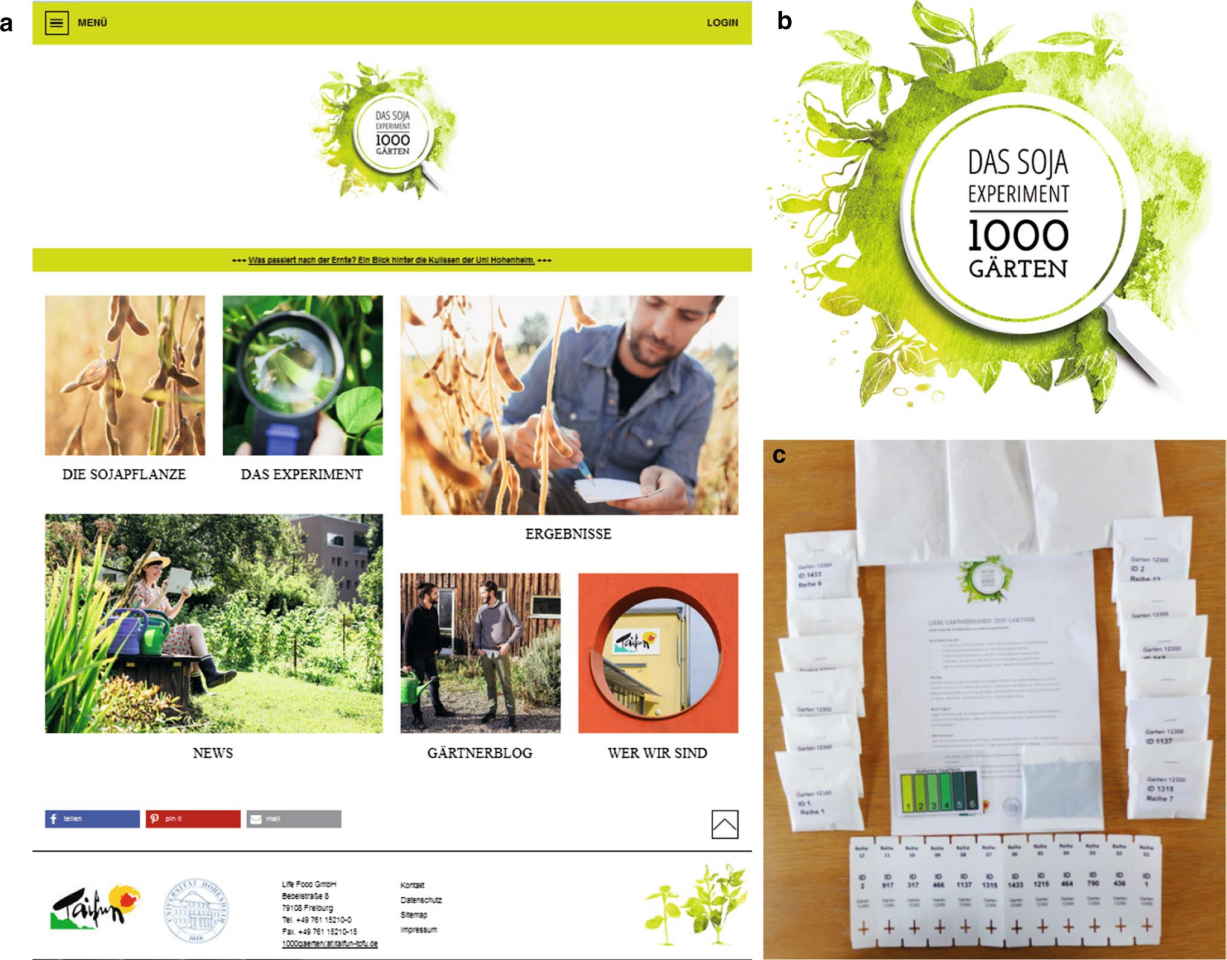
The soybean ‘1000 Gardens—the soybean experiment’ citizen science project (‘Die Sojapflanze’, Soybean; ‘Das Experiment’, The Experiment; ‘Ergebnisse’, Results; ‘Gärtnerblog’, Gardeners’ Blog; ‘Wer wir sind’, Who we are). **a** The 1000 Gardens project website (www.1000gaerten.de) and **b** logo of the project. **c** The starter pack sent to the participating citizens contained twelve bags with 100 seeds each, a bag with inoculum for Rhizobacteria inoculation, labels to mark the rows in the garden, a card to score the greenness of the leaves, and instructions on when and how to sow the seeds and to score the target traits

Prior to the start of the project, a website was designed to provide background information on soybean, the history of its cultivation, soybean for human consumption, as well as its positive effects for a sustainable agriculture and an optimal human diet (www.1000gaerten.de; Fig. 1a, b). The website provided guidance for participants on how to conduct the experiment and other sections including a blog for the citizen scientists to share their experiences and results. The project started in early 2016 with press releases and advertisements placed in several magazines on garden- or food-related topics, as well as on Taifun-Tofu products, accompanied by the launch of the website. Registration was open until the end of February 2016.

### Plant material

The soybean panel used in this study is comprised of 1710 breeding lines that can all be classified as early maturing material of maturity groups (MG) 000 and MG 00. The 1710 lines are derived from crosses among 20 parental lines of the same two maturity groups. The progeny from each cross were taken to the F_6_ generation by single-seed descent and were then continued as bulks. The parental combinations and the number of F_6:7_–F_6:9_ progenies in each cross are shown in Table S1. Some participants were sent 10 different breeding lines for evaluation (Experiment 1), and in the conditions of participation it was specified that the intellectual property rights of the breeding lines remained with the organizers of the study. The remaining participants were sent ten varieties (Experiment 2): ‘Alexa’, ‘Abelina’, ‘Merlin’, ‘Regina’ and ‘Sunrise’ of MG 000, ‘Lenka’, ‘Primus’, ‘Korus’, ‘Shouna’ and ‘Solena’ of MG 00. In addition, each participant got the two check varieties ‘Taifun3’ and ‘Adsoy’, where ‘Adsoy’ is an extremely early maturing genotype of MG 000 that might even be classified as MG 0000 and ‘Taifun3’ is slightly later maturing and from MG 000.

### Assessment of soybeans by the participants

The participants were sent instructions for the experiment, twelve bags with 100 seeds each for rows of 2 m length and a bag with inoculum for Rhizobacteria inoculation (Fig. 1c). Participants were asked to thin the germinated plants at a distance of 4 cm. Between rows a space of 0.5 m was recommended. The two common check cultivars were grown by each participant as the outermost rows, whereas the order of the ten breeding lines and cultivars followed the randomization.

Until harvest 16 morphological, physiological, and yield-related traits were listed for evaluation, including flowering, maturity, height, and pods per plant (Table 1). The participants entered the phenotypic data collected in their own gardens online into a database for scientific analysis.

**Table 1:** Description of the trait data

Nr.	Trait	Data type	Month^a^	Heritability Exp. 1^b^	Heritability Exp. 2^b^
1	Sowing date	Date	Apr., May		
2	Length of row	Metric (cm)	Apr., May		
3	Germination rate	Metric	May, June	0.40	0.99
4	Start of flowering	Date	June, July	0.28	0.63
5	Green value of the leaves	Nominal	July, Aug.		
6	Flower colour	Nominal	June, July		
7	End of flowering	Date	July, Aug.	0.42	0.80
8	Plant height	Metric (cm)	Aug., Sep.	0.69	0.98
9	Branching	Ordinal (w, m, s)^c^	July, Aug.	0.26	0.87
10	Distance lowest pod to ground	Metric (cm)	Aug., Sep.	0.55	0.96
11	Layers with pods	Metric	Aug., Sep.	0.41	0.94
12	Start yellowing of leaves	Date	Aug., Sep.	0.60	0.98
13	Lodging	Ordinal (1–9)	Aug., Sep.	0.32	0.92
14	Maturity date	Date	Aug., Sep.	0.60	0.96
15	Pods per plant	Metric	Aug., Sep.	0.28	0.84
16	Beans per 20 pods	Metric	Aug., Sep.	0.28	0.93
	*Quality traits scored centrally on returned beans*				
	Protein content	Metric (%)		0.81	0.99
	Oil content	Metric (%)		0.80	0.96

^a^Month when the trait is approximately scored

^b^Experiment 1 refers to the 1730 breeding lines and Experiment 2 to the 10 cultivars

^c^*w* weak, *m* medium, *s* strong

### Statistical analysis

Owing to the high number of participants, we performed two experiments. Experiment 1 included 1730 gardeners and was based on the 1710 breeding and their 20 parental lines, i.e. 1730 genotypes. It was laid out as an α-lattice design with ten replications, each replication with 173 incomplete blocks with a size of 10 plots. Here, each participant represented an incomplete block with 10 genotypes. Thus, the 1730 soybean lines were randomly assigned to one of 173 gardeners in ten zones, i.e. the ten replications, two of which were combined into five regions following latitude (Fig. S1). The reason for this was to ensure an even distribution of each genotype in north–south direction, as photoperiod and thus latitude is an important determinant of soybean adaptation and maturity (Kurasch et al. 2017). For Experiment 1, the model was $y_{ijk} = \mu + g_{i} + r_{j} + b_{jk} + \varepsilon_{ijk}$, where *y*_*ijk*_ is the observed phenotypic value of the *i*th genotype in the *k*th block of the *j*th replication, *µ* the mean, *g*_*i*_ the effect of the *i*th genotype, *r*_*j*_ the effect of the *j*th replication, *b*_*k*_ the effect of the *k*th incomplete block nested within the *j*th replication (= garden of a participant), and *ε*_*ijk*_ the residual error.

The remaining 762 participants were part of Experiment 2, where each participant was sent the same ten cultivars and each garden represented a randomized complete block. The model for the analysis of Experiment 2 was $y_{ij} = \mu + g_{i} + b_{j} + \varepsilon_{ij}$, where *y*_*ij*_ is the observed phenotypic value of the *i*th cultivar in the *j*th block, *µ* the mean, *g*_*i*_ the effect of the *i*th cultivar, *b*_*j*_ the effect of the *j*th block and *ε*_*ij*_ the residual error.

Variance components were estimated in full random models, while best linear unbiased estimates (BLUEs) were estimated with genotype as fixed effect. Heritabilities were estimated following the suggestion by Piepho and Möhring (2007). All statistical analyses were performed using the statistical software R (R Development Core Team 2014) and ASReml-R 3.0 (Gilmour et al. 2009).

## Results

### Participation of citizen scientists

**Fig. 2:**
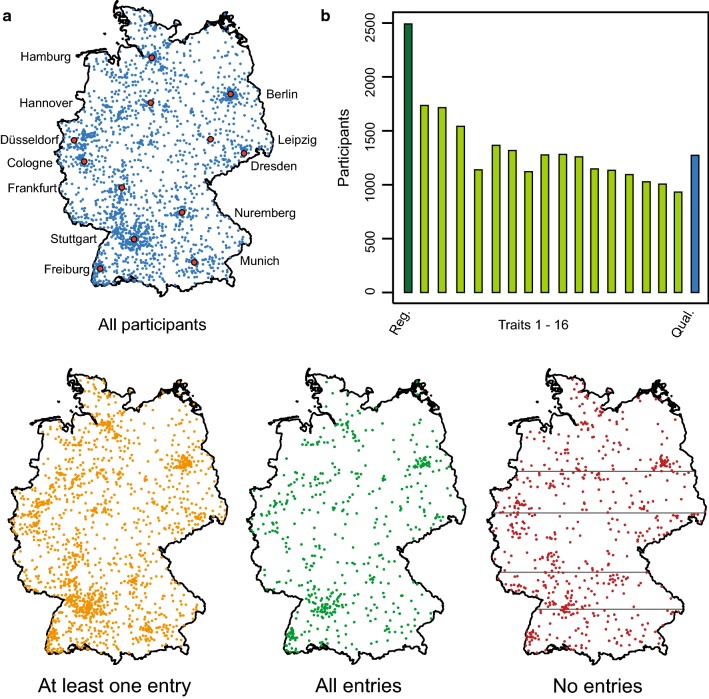
Geographic distribution of the participants and trait assessment. **a** The maps show Germany with all 2492 participants, those who scored at least one trait and entered it online in the database (*n* = 1790), those who scored all traits (*n* = 557), as well as those without any entry in the online database (*n* = 702). The grey horizontal lines in the latter indicate the latitudinal regions considered during randomization. **b** Number of participants scoring each of the 16 traits (the numbering refers to Table 1) and sending samples back for quality analysis (Qual.)

In total, 2492 citizen scientists volunteered to participate in the project (Fig. 2). The region in the south-west of Germany, particularly in the vicinity of the two cities Stuttgart and Freiburg, had more participants, also relative to their population, probably due to the two project partners being located there. Consequently, the initial press releases were more readily used for articles about the project in local newspapers and other media. This indicates that the number of participants in such an experiment can be controlled and thus increased through targeted media attention. Furthermore, Berlin also attracted a high number of participants compared to other major cities, probably due to its strong vegetarian and vegan scene. Obviously, people who are already interested in a topic are more open to participate, but the participants’ comments indicated that there was no obvious bias towards a particular group, such as vegetarians or environmental activists. Rather, the driving force behind participation was that people found the experiment exciting and were attracted to take part to learn something and contribute to science. An interesting option for future citizen science projects is to include social scientists or psychologists in order to investigate in more detail the motivation of the participants throughout the project, their backgrounds, as well as the consequences possibly drawn by the citizens from participating in a project.

### Quality of the data

The heritability of a trait expresses the fraction of the phenotypic variation that can be attributed to genotypic variation and can serve as a measure for the quality of the collected trait data. These heritabilities were high in the experiment with the twelve common cultivars but varied more strongly for the experiment based on the 1730 genotypes, ranging from 0.28 for the number of pods per plant to 0.69 for plant height (Table 1). The participants received instructions on how to assess the traits and were also reminded by emails around the time when the phenological stage to score each trait had arrived. However, the analysis also revealed that generally the quality of the data obtained by such an approach could be improved. The heritabilities of protein and oil content assessed on samples returned by the participants were higher than those of traits evaluated by the participants themselves, indicating that not so much the locations increased the error, but the variable evaluation by the non-trained gardeners. An example for this is flower colour, which despite being highly heritable was seen and scored quite differently by the participants, who, for example, scored a purple petal with a small white spot as either purple or as mixed coloured. Thus, additional instructions focusing on visualization of the tasks, for example, by online video clips or apps for smartphones, appear promising to improve compliance and accuracy of measurements in citizen science studies. Nevertheless, the results, for example the high heritability with 0.60 for maturity as a basis to study adaptive mechanisms, demonstrate the value of the citizen science approach. This approach has yielded a unique set of data, and phenotypic information from such a large number of environments throughout Germany could not have been collected using traditional approaches.

### Compliance of the citizen scientists

**Fig. 3:**
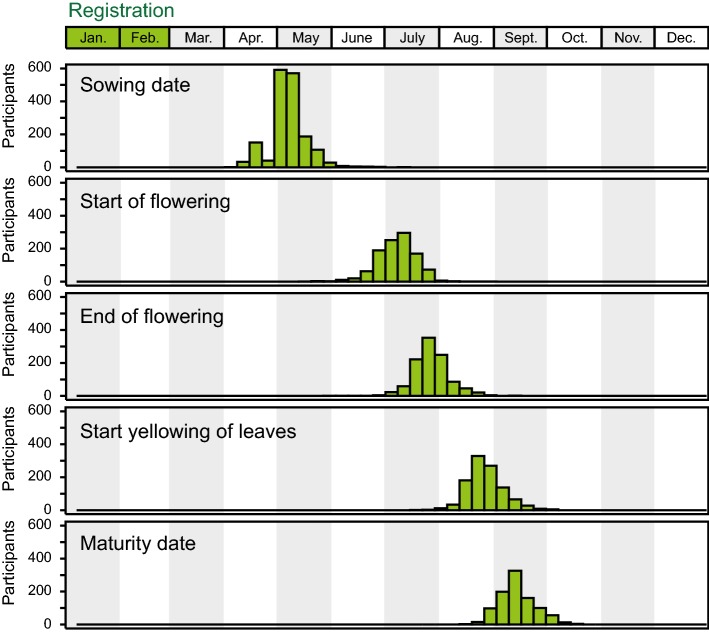
Time course of trait assessments. Dates when the different traits were assessed by the participants in 2016, illustrating the distribution of trait assessments throughout the course of the project

Of the initial 2492 gardeners who registered for the project and who were sent seeds, 30% (*n* = 757) never entered any data (Fig. 2). By comparison, Worthington et al. (2012) reported that for the Evolution MegaLab project 62% of the participants who registered did not submit a record. Thus, a certain loss of participants must be expected for this kind of approach, which must be taken into account when planning the project. In our study, this resulted in varying numbers of datapoints for each genotype, of which some were completely missing in some of the predefined latitudinal zones. This might have been prevented by a higher number of replications per genotype and zone. On the other hand, 1790 citizen scientists did collect data and entered them online. However, this number of gardeners entering data was further reduced during the project, sometimes due to adverse events like snails or hail destroying the plants, but also because the participants lost motivation and admitted that the required work was greater than they had expected (Fig. 2). The 16 trait assessments were designed to keep the participants motivated throughout the whole project (Fig. 3), and 57% (*n* = 1027) of the active gardeners recorded data until maturity, while 71% (*n* = 1273) even sent samples back for subsequent analysis of quality traits (Fig. 2).

**Fig. 4:**
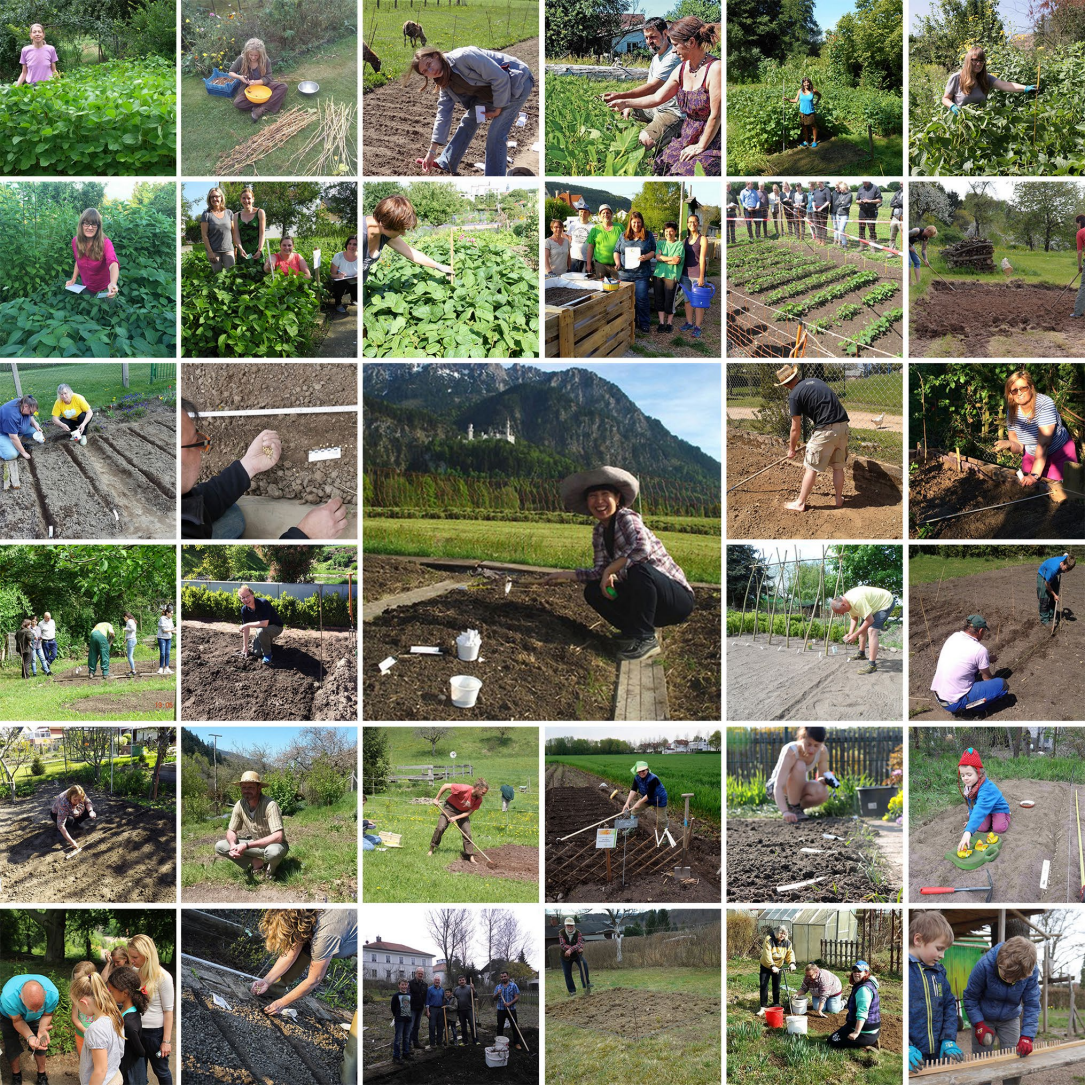
The citizen scientists at work. A collection of impressions of the citizen scientists at work, uploaded by themselves in the gardeners’ blog

A blog was established for the gardeners on the project website, which turned out to be frequently used by the participants (Fig. 4). By the end of 2016, there were 827 blog entries from 463 participants. The blog was used to share experience and results, but also stories beyond the experiment. The vast majority of gardeners who made blog posts gave a very positive feedback and indicated how much they enjoyed being part of the project and how much fun they had seeing soybeans grow in their gardens. It was thus an important instrument to create a sense of community for the participants, which probably also contributed to keeping them motivated. At the end of the project, a brochure was designed in which the scientific results and conclusions were shared with the citizen scientists (www.1000gaerten.de/aktivitaeten/ergebnisse).

### Media attention of the project

**Fig. 5:**
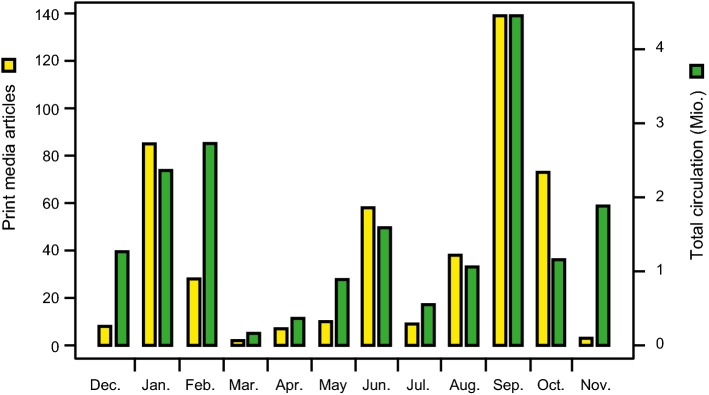
Attention of the 1000 Gardens project in print media. Articles covering the 1000 Gardens citizen science project in print media and their total circulation. The period from December 2015 to November 2016 is shown

From start to finish, the project attracted a great deal of media attention with articles and reports in print media, social media, radio, and TV. In print media, 461 articles appeared in 344 different newspapers and journals with a total circulation of almost 19 million, while online 235 articles were published (Fig. 5). In social media, the project was picked up 50 times, with a potential reach of about one million people. Moreover, 14 radio or TV reports covered the 1000 Gardens project. For all types of media, this attention covered the entire project duration. Although these numbers are estimates that are most likely to be incomplete, they do illustrate the great interest generated by the project in the media, which has attracted a much broader public than just the participating citizen scientists.

## Discussion

### Citizen science complements the toolbox for plant science studies

Citizen science is not a new approach, but for a long time has been employed by only few disciplines, mainly to survey birds and other organisms. In recent years, citizen science has gone beyond ecology and environmental sciences and citizen scientists are now also participating in projects on monitoring water quality, climate change or addressing evolutionary questions (Silvertown 2009). This broader use of the citizen science approach was also facilitated by the availability of internet and online databases. However, most plant science disciplines have so far not yet applied this approach, because researchers were either unaware or did not know how to exploit it for their own research.

Here, we present details on the 1000 Gardens project. Beside the aim to improve the soybean public image by communicating key messages about the multiple benefits of soybean to the public, the project had the objective to collect scientific data, mainly related to the adaptation of soybean. Clearly, such a data set with trait data from hundreds of locations throughout Germany would not have been possible by traditional means. We will next combine these phenotypic data with molecular data in order to decipher the genetic architecture underlying adaptation in early maturing soybeans and to investigate the effect of the environment on quality traits. Moreover, the project had a strong focus on plant breeding, as the genotypes evaluated by the citizen scientists were breeding lines from an applied soybean breeding programme. Based on the results from this project, we were able to identify lines that are well adapted to different regions in Germany where soybeans are not usually grown. They also show a good agronomic performance and have high protein contents, an important criterion for soybean cultivars. Thus, after additional yield trials in the respective regions, these lines have a high potential to become registered soybean varieties, either for animal feed or even food grade and destined for human consumption, if they possess tofu quality. This also illustrates the great potential of this approach in breeding of regional varieties, i.e. varieties that are tailored to the specific growth conditions but also the requirements of the processors, marketers, consumers and policy makers in a certain region. Instead of citizens as in our study, this might be better achieved by cooperating with farmers, who are able to also assess the yield potential of selected breeding lines and who are familiar with the needs of the different actors of the production chain(s) in their region. Notably, the cooperation with either citizens or farmers in plant breeding can be regarded as a participatory breeding approach, which is a valuable tool in developing countries to establish local varieties that are accepted by the farmers (Atlin et al. 2001). As illustrated by our example, citizen science and participatory breeding can be congruent approaches and depending on the project, a range of different combinations is possible.

Consequently, the presented 1000 Gardens project was a citizen science project that can be assigned to the scientific categories crop genetics and plant breeding. Both are disciplines that one would not intuitively associate with citizen science. Our successful project therefore demonstrates that the citizen science approach can be a valuable research tool for many research areas, also in plant sciences. A prerequisite is that researchers are willing to consider this approach, which may mean to leave well-trodden paths and to tackle their research questions from a different angle in order to make it compatible with the citizen science approach. Possible applications are, for example, the phenotypic evaluation of gene bank accessions under diverse climatic conditions in order to identify promising accessions for breeding, as well as to study adaptive mechanisms and genotype-by-environment interactions of crops or model species, thereby also demonstrating to the public the valuable work of gene banks in conserving this biodiversity. Particularly for vegetables the approach may prove valuable, as these are commonly grown by gardeners, who, in line with the basic idea of participatory breeding, might be involved when varieties with new characteristics or even entirely novel species are to be introduced into the market. Likewise, ornamentals appear predestined for the combined citizen science—participatory breeding approach, as they, too, are predominantly grown in gardens, on terraces or on balconies, by citizens who could score and also rate morphological and floral traits of breeding lines or yet not marketed species. The educational and scientific aspect in an ornamentals citizen science project might be the great insect die-off we are currently witnessing (Vogel 2017) and the identification of species or genotypes with a high attractiveness and nutritional quality for bees, butterflies, and other insects.

Future work should also address the question of the optimal experimental design for these kinds of studies, which, however, may vary depending on the particular set-up and the objectives of each project. Nevertheless, similar to the Mother and Baby trial design that meets the specific requirements of participatory breeding in developing countries by an iterative co-learning between farmers and researchers, the traditional experimental designs for plant breeding trials may not be the best for citizen science—participatory breeding studies as the one presented here (Bänziger and Diallo 2001; Atlin et al. 2002). As illustrated by the 1000 Gardens project, there is inevitably variation introduced by the mostly untrained participants. While this can be reduced by a better instruction and guidance of the participants, special attention should be paid to quality checks. An experimental design and statistical approaches that enable to assess the quality of the data of each participant would certainly be valuable in order to remove low-quality data and thereby improve the analysis of the entire data set and the conclusions drawn from it.

Our 1000 Gardens project was realized without a publicly funded project and a first grant proposal based on this experiment failed, as the reviewers were apparently unfamiliar with the concept of citizen science and were of the opinion that a traditional field trial would have been better. However, our results clearly show that even from a scientific point of view, this approach, which has not yet been used for most research areas, is attractive and can provide valuable and unique data sets. Thus, despite being novel and often without reference, funding bodies and organizations should open up to this kind of approach as a novel avenue to address scientific questions. Meanwhile, we were able to obtain funding for a second year of the 1000 Gardens project in 2018 through the German Federal Ministry of Education and Research (BMBF), which will allow to address additional research questions, for example genotype-by-year effects.

### The value of science and its public perception

The March for Science that took place on 22 April 2017 in hundreds of cities around the world was an important step for science (Abbott et al. 2017), but its outreach and perpetuity may be limited as it did not emotionally involve a broader public. Important questions are, therefore, (1) how the public perception of global challenges and the willingness to act can be improved and (2) how the work of scientists and the value of research and scientific evidence as essential components to secure our health, environment, safety, society, economies; in short, our future well-being can be explained to the broader public.

Among the various reasons for the inaction to major threats is that they are distant in time and space. The polar bear and its melting arctic home or sinking islands at some far-off places are poor symbols for the consequences of global warming as they convey the message that the threat is someplace and sometime else but not right here and now. An important element to bridge that psychological distance is therefore the reduction of a scientifically complex problem of rather amorphous nature to something that people can understand and have access to, for example by growing it in their garden. Furthermore, active engagement as part of a larger group can provide strength to the individual, who might not act on its own, as they become part of a community effort and joint achievements. We suggest that citizen science projects hold the potential to assist us in achieving this.

Our 1000 Gardens project illustrates that the experiment performed by the citizen scientists does not have to directly tackle the complex topic or challenge, but rather must attract peoples’ interest and be fascinating and entertaining to keep them motivated. It thereby allows to convey key messages that go beyond the actual experimental question. An important aspect is that the project provides a platform for discussion. Moreover, the projects may be designed to include children or young people as the future decision-makers. Similar to our study, the presented approach holds potential for a broad range of scientific topics or even to demonstrate the value of science itself.

Obviously, neither a single such project nor citizen science in general will stop climate change or save the world. However, citizen science can be a powerful scientific tool also for disciplines that so far have not considered this approach. In addition, such projects can be designed to educate people, but also to create a community feeling and movement that, in combination with the media attention, can influence policy makers and thus result in change. Thus, in future scientists may not only be marching in the streets, but instead be figuratively marching into people’s homes, thereby making the citizens scientists themselves.

## Conclusions

Taken together, we hope that this report on the 1000 Gardens citizen science project provides input for scientists on how to carry out and optimize citizen science projects and encourage others to exploit this potentially very powerful research tool for their own research.

## Electronic supplementary material

Below is the link to the electronic supplementary material.

Supplementary material 1 (PDF 302 kb)
