# Supplementary material for: The soybean experiment ‘1000 Gardens’: a case study of citizen science for research, education, and beyond
Source: Theor Appl Genet. 2018 Jul 3;132(3):617–26. doi: 10.1007/s00122-018-3134-2 (PMC6439140; doi:10.1007/s00122-018-3134-2)
Supplement: Supplementary file 1 — Supplementary material 1 (PDF 302 kb) [file 122_2018_3134_MOESM1_ESM.pdf]

The Soybean Experiment “1000 Gardens”: A case study of Citizen Science for research, education, and beyond

Tobias Würschum[1], Willmar L. Leiser[1], Felix Jähne[1], Kristina Bachteler[2], Martin Miersch[2], and Volker Hahn[1*]

[1] State Plant Breeding Institute, University of Hohenheim, 70593 Stuttgart, Germany

[2] Life Food GmbH, Taifun Tofuprodukte, 79108 Freiburg, Germany

✉ Volker Hahn, email: volker.hahn@uni-hohenheim.de

Supplementary Material

Table S1. Soybean breeding lines used in this study. The parents of the crosses are shown in the outer cells. Black numbers indicate biparental crosses between parents shown in the left column and the top row, red numbers display 3-way-crosses with parents from the left and right column and the top row, and blue numbers refer to 4-way crosses where all 4 parents in the outer cells are included.

	Taifun3	Primus	Protina	Gallec	Sigalia	Sultana	ESMentor	Merlin	Sirelia	Proteix	Amandine	
Taifun3							10	6				
Primus				1	83		33	48	7/11		8	Sultana
Protina		126		17	103					28/49		Primus
Gallec		106	122				28					Primus
Sigalia				243		94			6		2	Primus
Sultana		112										
ESMentor	6	17										
Merlin	26									79		
Sirelia		32								14		Protina
Proteix	7	24			39							Protina
Amandine		4	3						2			Primus
Aligator		9							16			
Paradis		16										
Korus		10							16			
Naya		17							20			
Solena		3							18			
Capnor		9							34			
1152							2					
Aveline									11	70		Protina
		Gallec		Proteix	Primus					Protina		

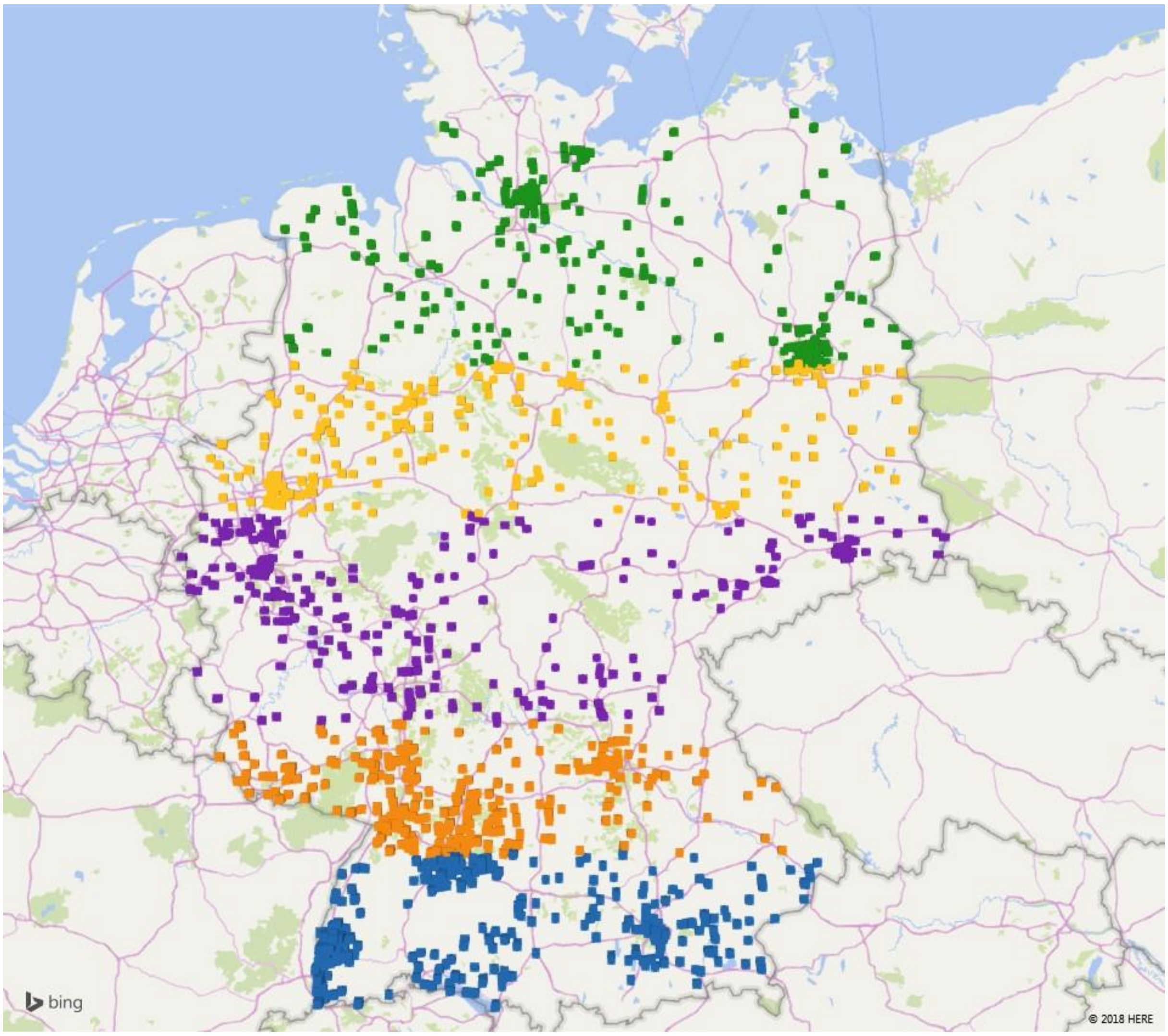

Fig. S1 Randomization of the participants of Experiment 1 into 10 replications of which two each formed 5 latitudinal zones.
